# Supplementary material for: District-Level Risk Factors for COVID-19 Incidence and Mortality in Nepal
Source: Int J Environ Res Public Health. 2022 Feb 24;19(5):2659. doi: 10.3390/ijerph19052659 (PMC8910479; doi:10.3390/ijerph19052659)
Supplement: Supplementary file 1 [file ijerph-19-02659-s001.zip › ijerph-1554155-supplementary.pdf]

Supplementary materials

District-level Risk Factors for COVID-19 Incidence and Mortality in Nepal

Dirga Kumar Lamichhane [1,*], Sabina Shrestha [2] and Hwan-Cheol Kim [1]

[1] Department of Occupational and Environmental Medicine, School of Medicine, Inha University, Incheon 22212, South Korea; carpediem@inha.ac.kr

[2] Department of Community and Global Health, Graduate School of Medicine, The University of Tokyo, Tokyo 113–0033, Japan; sabinashrestha2001@yahoo.com

* Correspondence: dirgalamichhane@gmail.com

Table S1. District-wise COVID-19 cases in Nepal (January 23, 2020 to January 22, 2021).

Country/ District	Province	Confirm ed cases[a]	Recovered cases[a]	Deceased cases[a]	Population projected 2021[b]	Rate of cases[c]	Rate of recovered cases[c]	Rate of deaths[c]
NEPAL		268948	263546	1986	30526143	881.042	863.345	6.506
Jhapa	One	5033	4993	23	939491	535.716	531.458	2.448
Terhathum	One	164	155	1	100495	163.192	154.237	0.995
Taplejung	One	161	160	0	130992	122.908	122.145	0
Udayapur	One	682	669	2	360705	189.074	185.470	0.554
Okhaldhunga	One	279	272	3	151762	183.840	179.228	1.977
Sankhuwasabha	One	399	392	3	155292	256.935	252.428	1.932
Panchthar	One	192	179	8	197440	97.245	90.660	4.052
Bhojpur	One	166	163	3	152216	109.056	107.085	1.971
Sunsari	One	9145	9111	26	931436	981.817	978.167	2.791
Khotang	One	224	220	0	169630	132.052	129.694	0
Ilam	One	495	482	5	314201	157.542	153.405	1.591
Dhankuta	One	278	269	5	171841	161.777	156.540	2.910
Morang	One	13236	13166	58	1108654	1193.880	1187.566	5.232
Solukhumbu	One	207	204	2	101726	203.488	200.539	1.966
Parsa	Two	3513	3449	50	729291	481.701	472.925	6.856
Mahotari	Two	2073	2041	29	719181	288.245	283.795	4.032
Saptari	Two	2187	2141	13	719000	304.172	297.775	1.808
Dhanusa	Two	3131	3097	29	852097	367.446	363.456	3.403
Sarlahi	Two	2861	2841	18	909891	314.433	312.235	1.978
Bara	Two	2182	2153	21	846772	257.684	254.260	2.480
Siraha	Two	2294	2270	20	711146	322.578	319.203	2.812
Rautahat	Two	2819	2785	30	862993	326.654	322.714	3.476
Nuwakot	Bagmati	1530	1456	17	288328	530.646	504.980	5.896
Rasuwa	Bagmati	238	227	1	45200	526.549	502.212	2.212
Kavre	Bagmati	3642	3584	33	404549	900.262	885.925	8.157
Dolakha	Bagmati`	802	772	10	186871	429.173	413.119	5.351
Dhading	Bagmati	1762	1742	8	355975	494.979	489.360	2.247
Sindhuli	Bagmati	660	631	4	312448	211.235	201.954	1.280
Bhaktapur	Bagmati	9245	9139	60	377660	2447.969	2419.902	15.887
Sindhupalchowk	Bagmati	1038	1010	16	294736	352.180	342.680	5.429
Chitwan	Bagmati	8065	7953	68	711629	1133.315	1117.577	9.556
Kathmandu	Bagmati	103523	100584	738	2300890	4499.259	4371.526	32.075
Makwanpur	Bagmati	4348	4290	42	466612	931.823	919.393	9.001
Lalitpur	Bagmati	16106	15806	69	585982	2748.549	2697.353	11.775
Ramechhap	Bagmati	590	567	19	209248	281.962	270.970	9.080
Baglung	Gandaki	1275	1241	7	285119	447.182	435.257	2.455
Parbat	Gandaki	546	489	28	148448	367.806	329.408	18.862
Gorkha	Gandaki	1550	1490	30	243272	637.147	612.483	12.332
Tanahu	Gandaki	1868	1835	13	348724	535.667	526.204	3.728
Syangja	Gandaki	1201	1184	10	246315	487.587	480.685	4.060
Kaski	Gandaki	7668	7544	20	597988	1282.300	1261.564	3.345
Lamjung	Gandaki	1142	1127	2	172158	663.344	654.631	1.162
Myagdi	Gandaki	302	278	2	110419	273.504	251.768	1.811
Manang	Gandaki	20	15	1	6284	318.269	238.702	15.913
Mustang	Gandaki	43	42	0	11240	382.562	373.666	0
Banke	Lumbini	5123	4960	45	621921	823.738	797.529	7.236
Palpa	Lumbini	2246	2214	2	246206	912.244	899.247	0.812
Kapilbastu	Lumbini	2314	2262	14	680984	339.802	332.166	2.056
Rupandehi	Lumbini	9708	9496	93	1091034	889.798	870.367	8.524
Gulmi	Lumbini	1244	1208	12	252731	492.223	477.979	4.748
Bardiya	Lumbini	2004	1927	50	486334	412.063	396.230	10.281
Pyuthan	Lumbini	1103	1076	15	243816	452.390	441.316	6.152
Arghakhanchi	Lumbini	1049	1023	4	202756	517.371	504.547	1.973
Rolpa	Lumbini	341	319	4	239145	142.591	133.392	1.673
Dang	Lumbini	4189	4119	6	660742	633.984	623.390	0.908
Jumla	Karnali	475	454	2	127214	373.387	356.879	1.572
Salyan	Karnali	665	656	5	276060	240.890	237.630	1.811

Surkhet	Karnali	2514	2492	13	426673	589.210	584.054	3.047
Jajarkot	Karnali	123	119	1	201913	60.917	58.936	0.495
Dolpa	Karnali	60	55	2	43058	139.347	127.735	4.645
Dailekha	Karnali	1666	1655	6	302085	551.500	547.859	1.986
Humla	Karnali	44	37	4	59800	73.579	61.873	6.689
Mugu	Karnali	37	35	2	65102	56.834	53.762	3.072
Kalikot	Karnali	295	292	3	162277	181.788	179.939	1.849
Doti	Sudurpachhim	1448	1444	1	213465	678.331	676.458	0.468
Baitadi	Sudurpachhim	735	706	3	267932	274.323	263.500	1.120
Dadeldhura	Sudurpachhim	793	776	2	160426	494.309	483.712	1.247
Kanchanpur	Sudurpachhim	1948	1929	4	539119	361.330	357.806	0.742
Darchula	Sudurpachhim	426	409	15	145761	292.259	280.596	10.291
Achham	Sudurpachhim	1761	1754	4	291468	604.183	601.781	1.372
Bajura	Sudurpachhim	733	724	5	158078	463.695	458.002	3.163
Kailali	Sudurpachhim	6111	6036	34	971320	629.144	621.422	3.500
Bajhang	Sudurpachhim	1115	1079	12	225304	494.887	478.909	5.326
Nawalparasi[d]	-	4742	4664	60	737968	642.575	632.006	8.130
Rukum[d]	-	381	360	15	231015	164.924	155.834	6.493

[a] Ministry of Health and Population. COVID-19 Update. Available from: https://covid19.mohp.gov.np/.

[b] National Population and Housing Census 2011 (Population Projection 2011–2031).

[c] Per 100,000 people.

[d] Nepal was divided into seven provinces in September 2015, and Nawalparasi and Rukum were split in two parts, ending up in two different provinces.

Table 2. Correlation coefficients among the variables.

Variables	(1)	(2)	(3)	(4)	(5)	(6)	(7)	(8)
(1) Population density	1.000							
(2) Household crowding	−0.055	1.000						
(3) No handwashing facilities	−0.081	−0.037	1.000					
(4) Obesity in women	0.579	−0.345	−0.177	1.000				
(5) Smoking in men	−0.078	0.037	0.248	−0.057	1.000			
(6) Percent of the elderly	−0.125	−0.065	−0.088	0.078	−0.053	1.000		
(7) Adult literacy	−0.345	0.558	0.044	−0.566	0.187	−0.235	1.000	
(8) Cases per 100,000 people	0.871	−0.106	0.001	0.714	−0.033	−0.060	−0.525	1.000

Table S3. Linear regression results with population density as the single explanatory variable.

Models	β	SE	t-value	*p* value	Lower 95%	Upper 95%
Model 1: Cases per 100,000						
Population density	0.949	0.064	14.92	<0.001	0.822	1.076
Intercept	247.96	42.80	5.79	<0.001	162.62	333.30
R-squared		0.759				
Model 2: Recovered cases per 100,000						
Population density	0.928	0.063	14.82	<0.001	0.803	1.053
Intercept	242.84	42.12	14.82	<0.001	158.85	326.83
R-squared		0.752				
Model 3: Deaths per 100,000						
Population density	0.006	0.0007	8.83	<0.001	0.005	0.007
Intercept	2.63	0.457	5.75	<0.001	1.72	3.54
R-squared		0.517				

SE, standard error.

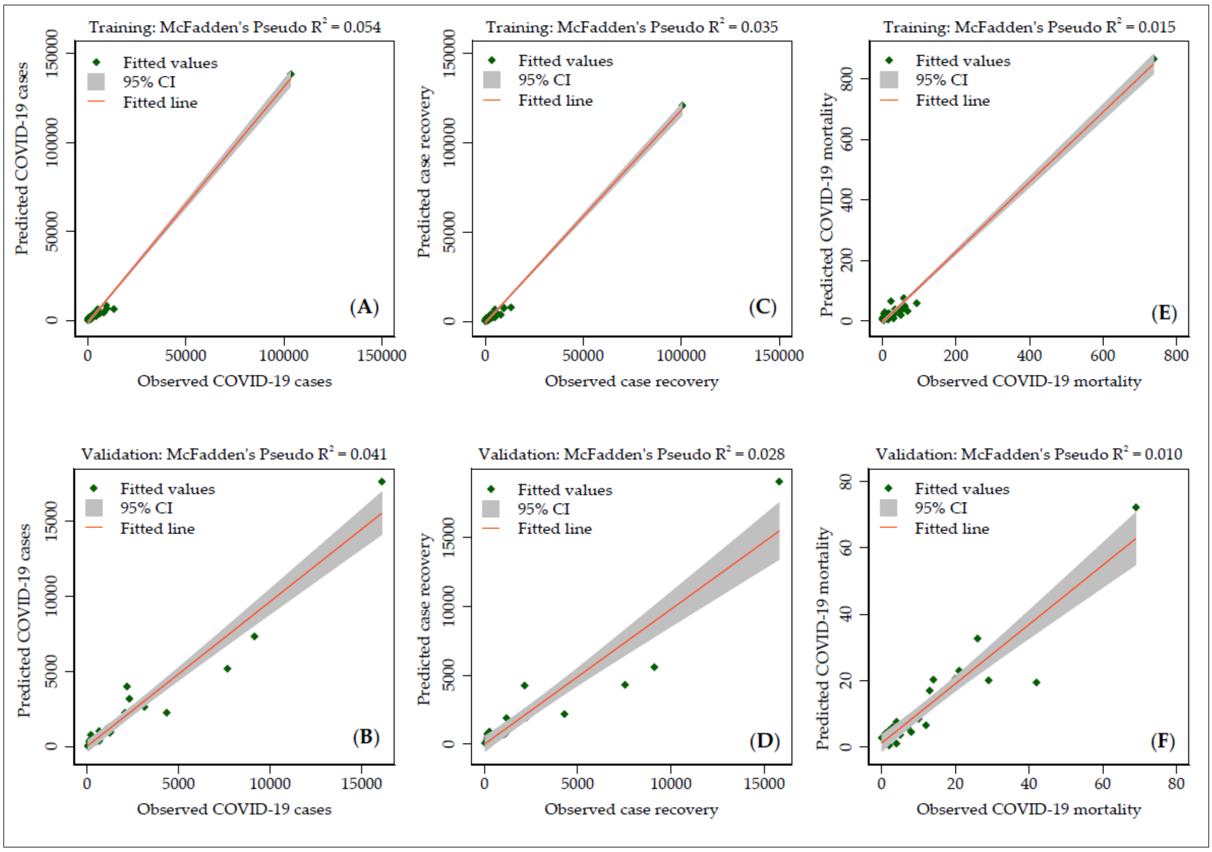

Figure S1. Scatter plot of observed and predicted COVID-19 cases (A and B), recovered cases (C and D), and mortality (E and F) for training and validation datasets using the negative binomial regression. R^2 is adjusted R^2 values.
